# Enabling Aqueous Processing of Ni‐Rich Layered Oxide Cathode Materials by Addition of Lithium Sulfate

**DOI:** 10.1002/cssc.202202161

**Published:** 2022-12-14

**Authors:** Marcel Heidbüchel, Thorsten Schultz, Tobias Placke, Martin Winter, Norbert Koch, Richard Schmuch, Aurora Gomez‐Martin

**Affiliations:** ^1^ MEET Battery Research Center Institute of Physical Chemistry University of Münster Corrensstr. 46 48149 Münster Germany; ^2^ Helmholtz-Zentrum Berlin für Materialien und Energie Hahn-Meitner-Platz 1 14109 Berlin Germany; ^3^ Forschungszentrum Jülich GmbH Helmholtz Institute Münster IEK-12 Corrensstr. 46 48149 Münster Germany; ^4^ Institut für Physik und IRIS Adlershof Humboldt-Universität zu Berlin Brook-Taylor-Str. 6 12489 Berlin Germany

**Keywords:** aqueous processing, cathode materials, energy storage, lithium-ion battery, lithium sulfate

## Abstract

Aqueous processing of Ni‐rich layered oxide cathode materials is a promising approach to simultaneously decrease electrode manufacturing costs, while bringing environmental benefits by substituting the state‐of‐the‐art (often toxic and costly) organic processing solvents. However, an aqueous environment remains challenging due to the high reactivity of Ni‐rich layered oxides towards moisture, leading to lithium leaching and Al current collector corrosion because of the resulting high pH value of the aqueous electrode paste. Herein, a facile method was developed to enable aqueous processing of LiNi_0.8_Co_0.1_Mn_0.1_O_2_ (NCM811) by the addition of lithium sulfate (Li_2_SO_4_) during electrode paste dispersion. The aqueously processed electrodes retained 80 % of their initial capacity after 400 cycles in NCM811||graphite full cells, while electrodes processed without the addition of Li_2_SO_4_ reached 80 % of their capacity after only 200 cycles. Furthermore, with regard to electrochemical performance, aqueously processed electrodes using carbon‐coated Al current collector outperformed reference electrodes based on state‐of‐the‐art production processes involving *N*‐methyl‐2‐pyrrolidone as processing solvent and fluorinated binders. The positive impact on cycle life by the addition of Li_2_SO_4_ stemmed from a formed sulfate coating as well as different surface species, protecting the NCM811 surface against degradation. Results reported herein open a new avenue for the processing of Ni‐rich NCM electrodes using more sustainable aqueous routes.

## Graphical abstract

**Keep dry**: Ni‐rich layered oxide cathodes are highly promising for high‐energy lithium‐ion batteries, and their aqueous electrode manufacturing is attractive regarding sustainability and recycling aspects. However, aqueous processing of these materials is challenging due to surface reconstruction. Here, it is shown that Li_2_SO_4_ as processing additive can protect the surface against water, leading to improved electrochemical performance.

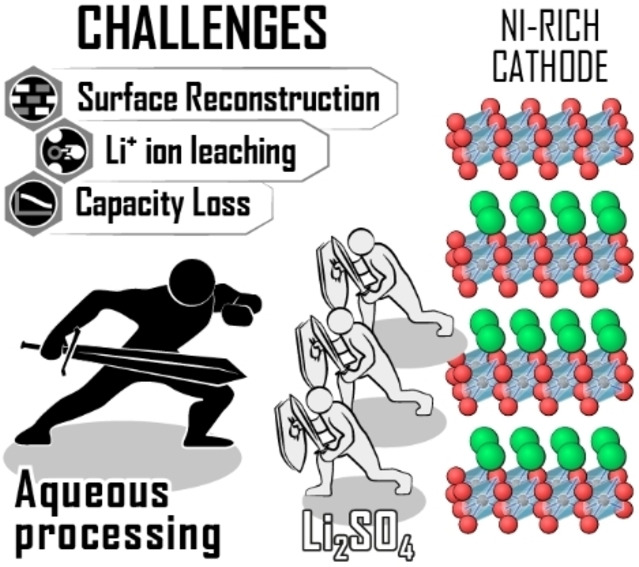

## Introduction

The positive electrode (cathode) is the major bottleneck of lithium‐ion batteries (LIBs) with regard to energy density and cost improvements.^[1]^ Furthermore, the production cost of the cathode material is responsible for >50 % of the overall material cost.[^1^, ^2^] Therefore, technological breakthroughs for an increased energy density and decreased production cost along the whole battery value chain are urgently needed.

State‐of‐the‐art (SOTA) cathode active materials (CAMs) are LiFePO_4_ (LFP) and layered oxides such as LiNi_1‐*x‐y*_Co_*x*_Mn_*y*_O_2_ (referred to as NCM*xyz*; *x*+*y*+*z*=1).[^3^, ^4^] By increasing the Ni content within NCM materials, the energy density on material level can be gradually increased.[^1^, ^5^] Since a higher Ni content (>80 % Ni) in layered transition metal oxides implicitly entails various challenges with respect to the material synthesis procedure, stability during electrode processing as well as life time, the broad commercialization of these CAMs still needs further advances.^[6]^ The SOTA processing route of Ni‐rich cathodes involves the use of fluorinated binders (i. e., polyvinylidene difluoride; PVdF) and often toxic organic solvents such as *N*‐methyl‐2‐pyrrolidone (NMP), which must be recovered during drying of the electrode.[^7^, ^8^] Water as non‐toxic and environmentally‐friendly processing solvent could make this additional recovery step unnecessary, reducing the electrode manufacturing cost and making the process more sustainable. Furthermore, recycling of LIBs, and especially of the cathode material, will become a highly relevant topic in the coming years.^[9]^ The conversion of electrodes into black mass, which typically consists of active material, binder, and conductive additives, is expected to be easier and cheaper for aqueously‐processed cathodes (e. g., by using fluorine‐free binders).^[10]^

However, Ni‐rich NCM CAMs are highly sensitive towards moisture, inevitably leading to surface reconstruction and formation of surface species accompanied by lithium leaching upon contact to water during processing or exposure to ambient atmosphere (e. g., Li_2_CO_3_ or Ni‐based carbonates/hydroxides), which deteriorate cell performance and cycle life.[^11^, ^12^, ^13^] When exposed to water, Li^+^/H^+^ exchange can take place, which leaches Li^+^ from the bulk of the NCM material and dissolves into the electrode paste dispersion during processing. Besides the loss of electrochemically active Li, the Li^+^/H^+^ exchange shifts the pH of the electrode dispersion to higher (basic) values. The extent of the Li‐leaching, which leads to a rapid rise of the electrode paste pH, is significantly influenced by the Ni content in Ni‐rich NCMs as well as the solid content in the electrode dispersion, that is, cathode materials with high Ni content are more prone to surface degradation.^[14]^ An electrode paste with a pH value >9 reacts with the Al_2_O_3_ passivation layer of the Al current collector, leading to a breakage/removal of the passivation layer. Afterwards, the bare Al is subject to anodic dissolution accompanied by the release of gaseous H_2_, resulting in the formation of large pits in the composite electrodes as well as the current collector foil below, which have a negative impact on the mechanical stability and integrity of the electrodes.^[15]^

A commonly taken approach to suppress current collector corrosion lies in the protection of the Al current collector by carbon coating^[16]^ or by using dilute acids to decrease the pH of the paste during electrode manufacturing.[^17^, ^18^] The latter strategy, especially when using phosphoric acid, might lead to the in‐situ formation of transition metal or lithium‐containing phosphate coatings at the surface of cathode particles. This coating could protect the CAM from further degradation in contact with water and stabilize the electrochemical performance of aqueously‐processed electrodes. In addition, the positive impact of surface coating by annealing with lithium phosphate (Li_3_PO_4_) on the electrochemical performance of Ni‐rich layered oxides has been recently reported.^[19]^ It is worth to mention that coatings, which can be applied without an additional annealing step, are able to reduce the production cost of the cathode and the overall manufacturing time.^[20]^ It is expected that the sulfate anion (SO_4_^2−^) reacts similarly to PO_4_^3−^, leading to the formation of a protective surface film;[^21^, ^22^] however, such a study combined with aqueous processing has not been reported so far. Besides “direct” pH modification, the addition of Li salts [e. g., lithium bis(trifluoromethanesulfonyl)imide; LiTFSI] has proven to mitigate the pH rise and the Li loss upon electrode processing, therefore, for example, enabling aqueous processing of the high‐voltage spinel cathode LiNi_0.5_Mn_1.5_O_4_.^[2]^

In this work, lithium sulfate (Li_2_SO_4_) is studied as a low‐cost additive (from 1 to 5 wt %) for aqueous electrode processing of Ni‐rich NCM811 cathodes. The impact of Li_2_SO_4_ addition on the material, electrode, and electrochemical properties is herein systematically evaluated. First, the impact of exposure of NCM811 to water and the beneficial influence of the Li_2_SO_4_ additive was evaluated with regard to surface (morphology and surface area) and bulk properties (lithium loss and Li^+^/Ni^2+^ cation mixing disorder). Aqueously processed electrodes were then investigated regarding their thermal stability, adhesion to the Al current collector foil, and through‐plane electronic conductivity. The use of a carbon‐coated Al current collector foil was also explored to avoid corrosion of the current collector due to the alkaline slurry pH value. Finally, the electrochemical performance for aqueously processed electrodes was compared with conventional NMP‐processed electrodes in NCM811||graphite full cells. Results reported herein prove a simple and effective method to enable the processing of Ni‐rich layered oxide cathode materials using aqueous routes by the addition of Li_2_SO_4_.

## Results and Discussion

### Impact of water exposure on NCM powder properties

**Figure 1:**
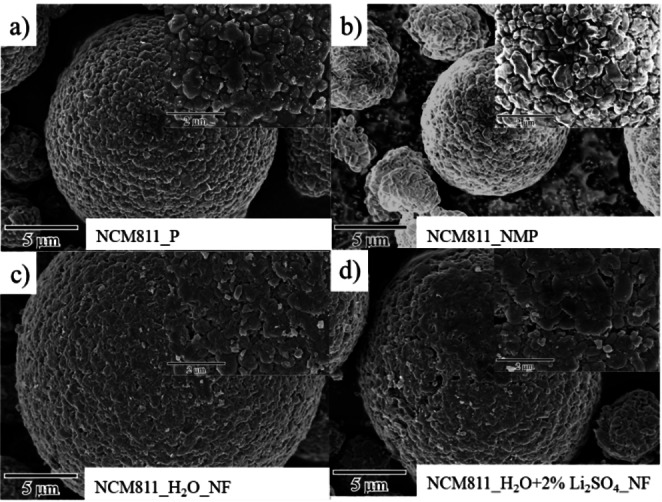
SEM images of (a) pristine NCM811 and after dispersion for 1 h at 15000 rpm in (b) NMP, (c) de‐ionized water, and (d) de‐ionized water with 2 wt % Li_2_SO_4_ ⋅ H_2_O. After dispersion, the unfiltered (NF) NCM811 was dried at 80 °C overnight and then mortared.

In an effort to investigate the detrimental impact on material characteristics when NCM811 is in contact with water, the particle morphology of the active material was first investigated after 1 h dispersion in water with/without 2 wt % Li_2_SO_4_ ⋅ H_2_O addition (based on the overall solid content) and compared to conventional NMP‐based processing. As can be seen in scanning electron microscopy (SEM) images shown in Figure 1a, pristine secondary particles are spherical with a diameter of around 10 μm and a defined surface composed of granular primary particles. No significant morphological changes were observed when using NMP as dispersing solvent (Figure 1b). After water exposure (Figure 1c,d), there are changes in the surface appearance of cathode particles. Li leaching from NCM811 leads to the formation of a surface‐film on the active material when exposed to water for 1 h, which can likely be attributed to the formation of Li_2_CO_3_ or NiOOH‐like species.^[12]^ Likewise, the re‐precipitation of Li_2_SO_4_ causes a pronounced surface smoothing, which resembles a surface coating (Figure 1d), similarly to previous works on Li_3_PO_4_.[^23^, ^24^] After exposure to water and the addition of Li_2_SO_4_, grain boundaries between primary particles are no longer well defined.

It is well‐known that Li leaches from the near‐surface region of NCM811 into the water during aqueous processing.^[14]^ During the drying step at 80 °C, Li is re‐deposited at the particle surface in the form of Li salts (e. g., Li_2_CO_3_) when no filtration (NF) step is used. As a result, the overall Li content within the cathode material can remain nearly unchanged. However, it is very important to differentiate between the Li from the bulk, which is electrochemically active, and the residual Li species formed at the surface of cathode particles, which are electrochemically inactive and degrade the electrochemical performance.^[25]^ To ensure an appropriate differentiation the NCM811 powders were filtrated after water exposure, and the stoichiometry was also determined by inductively coupled plasma optical emission spectrometry (ICP‐OES, Table 1). After 1 h dispersion in water (solid content of 50 wt %), roughly 2 % of the Li from the layered structure is converted into water‐soluble Li‐residues, which could be probably found in the filtrate, as demonstrated for the filtrated (F) samples compared to the not filtrated (NF) ones. However, the ICP‐OES results indicate the same Li content after filtration, both for samples with and without use of Li_2_SO_4_ ⋅ H_2_O. The Li leaching from the bulk into the water results in an increase of the Brunauer–Emmett–Teller (BET) specific surface area (Table 1). The BET surface area increase of the water‐exposed CAM could be notably suppressed by addition of Li_2_SO_4_ ⋅ H_2_O, which is probably related to re‐precipitation of the sulfate species at the newly generated surfaces.

**Table 1:** Stoichiometry determined by ICP‐OES, BET specific surface area, and calculated Li^+^/Ni^2+^ disorder (from XRD analysis) of pristine NCM811 (NCM811_P) and NCM811 materials after dispersion in NMP (NCM811_NMP) or de‐ionized water (NCM_H_2_O) with/without addition of 2 wt % Li_2_SO_4_ ⋅ H_2_O, either filtrated (F) or not filtrated (NF).

Sample	Stoichiometry	BET surface area [m^2^ g^−1^]	Li^+^/Ni^2+^ disorder [%]
NCM811_P	Li_0.97_Ni_0.80_Co_0.10_Mn_0.10_O_2_	0.2	2.30
NCM811_NMP	Li_0.97_Ni_0.81_Co_0.10_Mn_0.10_O_2_	0.4	3.40
NCM811_H_2_O_NF	Li_0.96_Ni_0.81_Co_0.10_Mn_0.09_O_2_	1.9	2.46
NCM811_H_2_O_F	Li_0.94_Ni_0.81_Co_0.10_Mn_0.10_O_2_	–	–
NCM811_H_2_O+2 % Li_2_SO_4__NF	Li_0.99_Ni_0.81_Co_0.10_Mn_0.09_O_2_	1.1	2.58
NCM811_H_2_O+2 % Li_2_SO_4__F	Li_0.94_Ni_0.81_Co_0.10_Mn_0.10_O_2_	–	–

X‐ray diffraction (XRD) measurements of the NCM811 powders were performed along with Rietveld refinements to evaluate changes in crystallinity and degree of cation mixing for the different materials regarding the processing conditions. XRD patterns and detailed Rietveld refinements of the different NCM materials after water exposure with/without Li_2_SO_4_ ⋅ H_2_O are shown in Figure S1 and Table S1. All samples adopt a hexagonal α‐NaFeO_2_ structure with the $R\bar{3}m$ space group. The obvious splitting of (006)/(102) and (108)/(110) reflections indicates a good hexagonal ordering within the structure. Neither significant changes in structural parameters nor Li/Ni cation mixing disorder are observed. As Li is likely leached from the near‐surface region of the CAM, no significant changes in the bulk can be detected by XRD. Nevertheless, a slightly increased cation disorder was found for all samples compared to the pristine material. By combining the ICP‐OES results with the BET surface area and XRD results, one can conclude that the addition of Li_2_SO_4_ ⋅ H_2_O can provide an enhanced protection against water exposure and probably suppresses surface reconstruction and Li leaching, but only in a small amount, which is not detectable by ICP‐OES.

**Figure 2:**
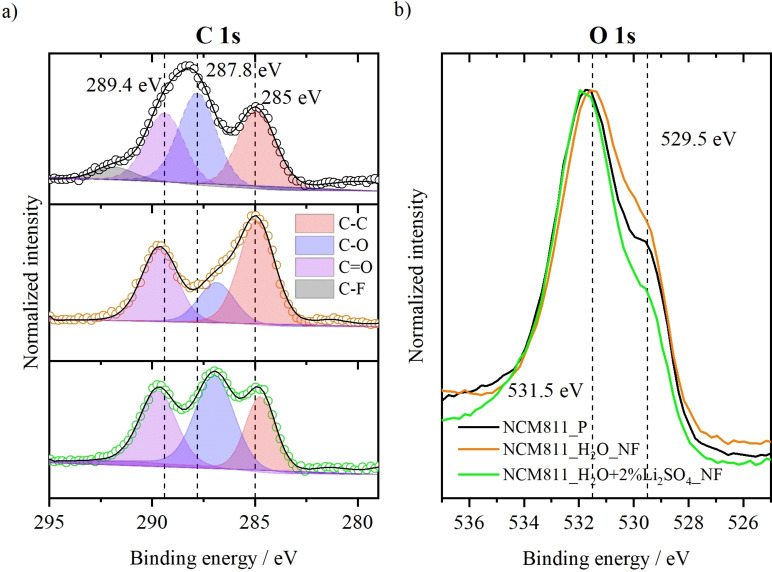
(a) C 1 s and (b) O 1 s XPS core‐level spectra of the NCM 811 CAMs for different processing methods.

Thermogravimetric analysis (TGA) indicates formation of different surface species after contact with water or Li_2_SO_4_ (Figures S2 and S3). In addition to the TGA results the surfaces of these powdered materials were analyzed using X‐ray photoelectron spectroscopy (XPS) (Figure 2). First of all, the presence of sulfur after washing with Li_2_SO_4_ indicates the formation of a coating on the NCM surface (Figure S4). In addition, the surface of NCM is also altered by the processing method. The C 1s core‐level spectra (Figure 2a) show two peaks at around 285 and 287 eV corresponding to C−C and C−O bonding, which are probably related to carbon residues and their oxidation under air.^[14]^ Moreover, another peak around 290 eV corresponds to C=O bonding and indicates the presence of carbonates (e. g., Li_2_CO_3_) after contact with air or water.^[14]^ After 1 h dispersion in water, the area of C−O peak decreases and shifts from 287.6 to 286.7 eV, potentially related to the formation of different C−O species (e. g., transition metal carbonates or carbonyl compounds) after contact with water.^[26]^ The area of this C−O peak again increases after addition of Li_2_SO_4_. This would indicate a reaction of NCM (or the surface impurities) with Li_2_SO_4_ in an aqueous media, which would lead to the formation of transition metal carbonates. In the O 1s core‐level spectra (Figure 2b), the metal–oxide (M−O) peak is visible around 529.5 eV. The other peak around 531.5 eV can be attributed to surface residues (e. g., LiOH/Li_2_CO_3_/Li_2_O), which are impurities from the synthesis, storage, and processing method. After 1 h dispersion in water, the M−O bonding is more pronounced, which indicates cleaning of the NCM surface from the contaminants.^[14]^ However, after addition of Li_2_SO_4_, the intensity of the M−O peak decreases again. Here, one can expect that the surface is covered with a sulfate coating instead of surface impurities that decreases the intensity of the M−O bonding.

### Impact of aqueous processing on NCM811 cathode properties

The SOTA processing method of cathodes involves a polyvinylidene difluoride (PVdF) binder, which is dissolved in NMP as processing solvent during electrode preparation. Since PVdF is insoluble in aqueous media, water‐soluble binders, such as sodium‐carboxymethyl cellulose (Na‐CMC)^[27]^ or polyacrylic acid (PAA), are promising binders for aqueous processing of Ni‐rich NCM materials.^[15]^ Furthermore, a common approach to improve electrode properties such as binding strength lies in the use of a second binder (e. g., acrylate latex binder).[^18^, ^28^, ^29^] In this work, a combination of Na‐CMC (1.5 wt %) and an acrylate‐based binder (ENEOS acrylate binder, ENEOS Corporation; 1.5 wt %) was used for the aqueous processing of NCM811 and compared to reference electrodes processed by PVdF and NMP.

**Figure 3:**
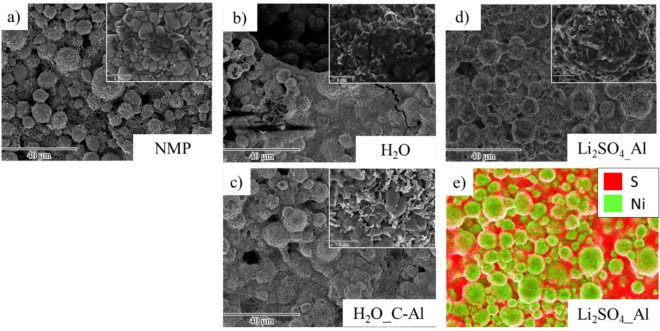
SEM images showing the morphology of NCM811 cathodes processed (a) with NMP, (b) with water on Al foil as current collector, (c) with water on carbon‐coated Al foil (C−Al) as current collector, (d) with 2 wt % Li_2_SO_4_ and water on Al foil as current collector. (e) Elemental distribution of Ni (green) and S (red) with 2 wt % Li_2_SO_4_ and water on Al foil as current collector.

Figure 3 shows the SEM analysis of the (a) NMP‐processed, (b,c) water‐processed, and (d) water‐processed electrodes with addition of Li_2_SO_4_ ⋅ H_2_O using either bare Al foil (Al) or carbon‐coated Al foil (C−Al), respectively. The smoothing of the NCM surface (Figure 1) after contact with water and/or lithium sulfate is also visible in the electrodes (inset Figure 3b–d). The Li leaching, Li^+^/H^+^ exchange, and thus pH increase (>9) of the electrode paste leads to dissolution of the Al_2_O_3_ passivation layer from the Al current collector. Afterwards, the bare Al reacts with the electrode paste, leading to anodic dissolution Al accompanied by H_2_ gas evolution,^[30]^ forming bubbles within the wet coating films. As a result, large cracks/pits (>20 μm in diameter) can be seen in the dried electrode (Figure 3b).^[31]^ SEM images shown in Figure 3 show significant differences between the two aqueously processed coatings compared to the NMP‐processed ones. While the NMP‐processed cathodes do not exhibit any cracking of the coating, the aqueously processed electrodes show evident holes on the surface, being more visible for the electrodes processed without Li_2_SO_4_ ⋅ H_2_O (Figure 3b). In contrast, by using carbon‐coated Al foil as current collector, the corrosion of Al foil and the formation of cracks in the surface of the coatings can be significantly suppressed (Figure 3c). An energy‐dispersive X‐ray spectroscopy (EDX) mapping study of Li_2_SO_4_ containing electrodes (Figure 3e) indicates a homogeneous re‐precipitation of Li_2_SO_4_ on the NCM surface as well as in the conductive carbon‐binder domain.

The pH value of the electrode paste after aqueous processing using a high‐energy disperser for 1 h was around 11.44, which was slightly reduced to around 11.15 after addition of 2 wt % Li_2_SO_4_ ⋅ H_2_O (Table 2). With higher amounts of Li_2_SO_4_ ⋅ H_2_O within the electrode paste (i. e., 5 wt %), the pH decreases to 11.05, which, however, is still far above the threshold for avoiding Al foil corrosion (pH>9),^[16]^ and therefore all coated electrodes are prone to current collector corrosion due to the high pH of the electrode pastes. Al corrosion could only be suppressed in this case by using a carbon‐coated Al current collector.

**Table 2:** pH values of the NCM811 cathode pastes after dispersion, adhesion force, and resistance of the prepared NCM811 electrodes (without pressing) using either pure aluminum foil (Al) or carbon‐coated Al foil (C−Al) as current collector.

Electrode	pH	Adhesion force [N cm^−2^]	Resistance [Ω]
NMP	–	184	1.2
H_2_O_Al	11.44	208	1.5
H_2_O_C‐Al	11.44	220	1.3
H_2_O+2 % Li_2_SO_4__Al	11.15	230	3.5
H_2_O+2 % Li_2_SO_4__C‐Al	11.15	227	3.2
H_2_O+5 % Li_2_SO_4__Al	11.05	211	5.9
H_2_O+5 % Li_2_SO_4__C‐Al	11.05	198	4.4

In addition, the adhesion of the electrode coating to the current collector might have a critical influence on the electrochemical properties. The adhesion force as well as the through plane electronic conductivity of the coated electrodes (using bare Al foil or carbon‐coated Al foil as current collector) were measured, as shown in Table 2. The mechanical stability of the electrode is also affected by aqueous processing. Even though the aqueously processed electrodes show large pits, the adhesion between current collector and electrode coating is higher for the aqueously processed electrodes than the PVdF/NMP‐reference, which might be related to the use of the different binder system (Na‐CMC/acrylate). By addition of Li_2_SO_4_ ⋅ H_2_O to the electrode paste, the adhesion force of these electrodes is even higher. This might be related to the better network between conductive carbon, binder, and NCM811 particles with only minor pits within the electrode, as confirmed by SEM analysis. As expected from the more homogeneous electrode morphology (Figure 3), the adhesion force with the use of carbon‐coated Al foil as current collector is similar or higher for most cathode formulations (Table 2).

The through‐plane resistance of the electrodes is depicted in Table 2 and Figure S6. The aqueously processed electrodes without Li_2_SO_4_ ⋅ H_2_O have only slightly higher resistances than the PVdF/NMP‐processed electrode. By using carbon‐coated Al foil (C−Al), the resistance could be decreased from 1.5 to 1.3 Ω. However, the Li_2_SO_4_ ⋅ H_2_O itself acts as an electronic insulator and the resistance increases with rising amount of Li_2_SO_4_ ⋅ H_2_O within the electrode from 2 to 5 wt %. Furthermore, the positive impact or rather the improved electronic conductivity of the carbon‐coated Al foil is particularly visible for electrodes prepared with 5 wt % Li_2_SO_4_ ⋅ H_2_O.

### Electrochemical performance of aqueously processed cathodes in NCM811||graphite full cells

The electrode processing method not only influences the physical electrode properties but also the resulting electrochemical behavior. Initially, the specific capacity of the NCM811 materials, as well as the rate capability and stability at increasing upper operating cell voltages (4.3 vs. 4.4 vs. 4.5 V) was evaluated in NCM811||Li metal cells, as shown in Figure S7. During the C‐rate study, the specific discharge capacity increases by using carbon‐coated Al foil and by rising the amount of Li_2_SO_4_ ⋅ H_2_O added during electrode paste processing (at 4.3 V), while the stability at higher cell voltages (4.4 and 4.5 V) is also improved. The rate capability of the aqueously processed electrodes by using Li_2_SO_4_ is only slightly inferior to the reference NMP‐processed electrodes. The contact time between NCM and water also influences the electrochemical performance (Figure S7). By decreasing the dispersion time from 60 to 20 min, the capacity of electrodes without addition of Li_2_SO_4_ ⋅ H_2_O increases, which is probably related to a lower extent of Li leaching and surface reconstruction. However, decreasing the contact time has only minor influence on the electrochemical performance of Li_2_SO_4_ ⋅ H_2_O containing cathodes, indicating protection against water caused by the Li_2_SO_4_ ⋅ H_2_O coating on the NCM surface.

**Figure 4:**
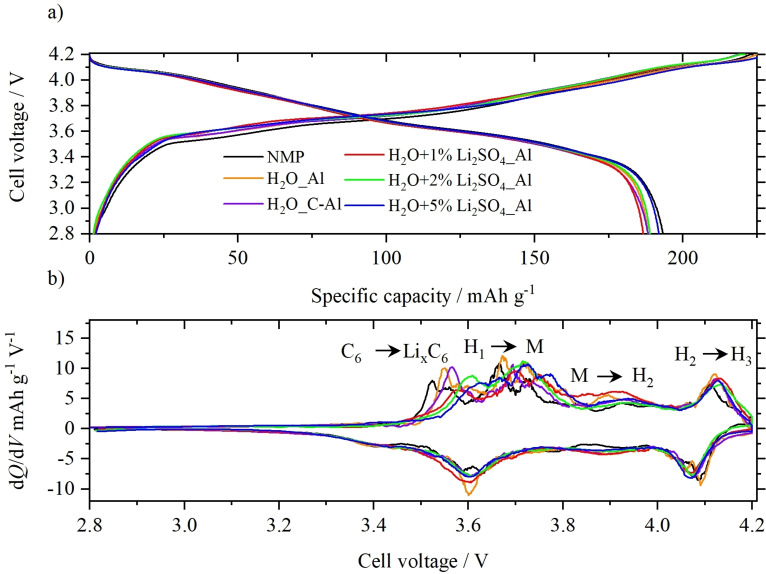
First cycle (a) cell voltage profiles and (b) corresponding differential capacity (d*Q*/d*V*) vs. cell voltage plots for NCM811||graphite cells using different cathode processing methods at 0.1 C [two‐electrode configuration; N/P ratio: 1.15 : 1.00; electrolyte: 1 m LiPF_6_ in 3 : 7 vol % ethylene carbonate (EC)/ethyl methyl carbonate (EMC)+2 wt % vinylene carbonate (VC)]. 1st cycle *C*_Eff_ can be found in Table S2.

Long‐term electrochemical evaluation of Ni‐rich cathode materials under realistic testing conditions was performed in NCM811||graphite full‐cells within the cell voltage range 2.8–4.2 V in a two‐electrode configuration.^[33]^ Figure 4a,b show the 1st cycle cell voltage profiles and differential capacity (d*Q*/d*V*) vs. cell voltage plots of all differently processed cathodes in NCM811||graphite cells at 0.1 C, respectively. The cell voltage profile (Figure 4a) indicates an influence from the processing method on the (de‐)lithiation processes. The d*Q/*d*V* vs. voltage plot enables a more detailed investigation of the (dis)charge reaction. Generally, the d*Q*/d*V* plot of NCM811||graphite full cells shows four different redox activities during charge. The first redox peak at around 3.5 V is related to Li^+^ intercalation into the graphite anode.^[34]^ Further charging leads to oxidization (=de‐lithiation) of NCM811 with transitions from hexagonal (*H*) phases to monoclinic (*M*) phases.^[34]^ The cells using NMP‐processed cathodes exhibit the lowest over‐potential for Li^+^ intercalation into the graphite anode, followed by cells containing positive electrodes without using Li_2_SO_4_ ⋅ H_2_O. The charge voltage profile of cells using cathodes without Li_2_SO_4_ ⋅ H_2_O shows only a minor influence from the current collector used. In contrast, cells comprising cathodes processed with Li_2_SO_4_ ⋅ H_2_O show the highest overpotential during charge. This indicates a de‐lithiation barrier, which is probably related to the lithium sulfate containing surface coating. By increasing the amount of Li_2_SO_4_ ⋅ H_2_O within the electrode, this overpotential increases gradually. Interestingly, the 1st cycle discharge capacity, however, increases with increasing amount of Li_2_SO_4_ ⋅ H_2_O within the cathode. By using 5 wt % of Li_2_SO_4_ ⋅ H_2_O instead of 2 wt %, the discharge capacity increases from around 189 to 192 mAh g^−1^ (see Table S2). The 1st cycle coulombic efficiency (C_Eff_) of the aqueously processed electrodes without Li_2_SO_4_ ⋅ H_2_O slightly increases by using carbon‐coated Al foil from 84.5 % (orange) to 85.5 % (purple curve). In addition to carbon‐coated Al foil as current collector, the highest first cycle C_Eff_≈85.6 % is achieved for 2 wt % of Li_2_SO_4_ ⋅ H_2_O. With further amount of Li_2_SO_4_ ⋅ H_2_O in electrodes on bare aluminum foil, the first cycle C_Eff_ decreases again. However, compared to the NMP ‐processed electrodes, the discharge capacity as well as the first cycle C_Eff_ (≈86.5 %) are inferior for the aqueously processed electrodes. During the initial 100 cycles, the C_Eff_ of each cathode increases up 99.9 % with slightly better values by using carbon‐coated Al foil (Figure S8). It is worth to mention, that the C_Eff_ of aqueously processed electrodes with Li_2_SO_4_ ⋅ H_2_O on Al foil increases more slowly than for the electrode without Li_2_SO_4_ ⋅ H_2_O. However, after 50 cycles the C_Eff_ of aqueously processed electrodes with Li_2_SO_4_ ⋅ H_2_O surpasses the aqueous‐processed reference.

**Figure 5:**
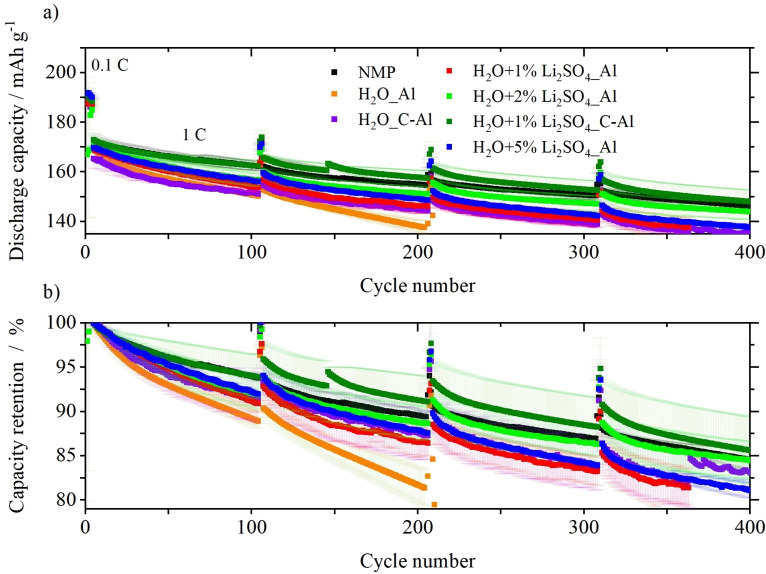
(a) Specific discharge capacity and (b) capacity retention (calculated on the basis of the 5th cycle discharge capacity) of the long‐term cycling stability experiments in NCM811||graphite full cells at a rate of 1 C. Standard deviation was calculated from at least three different cells. Cell voltage range: 2.8–4.2 V, N/P ratio: 1.15 : 1.00, electrolyte: 1 m LiPF_6_ in 3 : 7 vol % EC/EMC+2 wt % VC.

The positive impact of Li_2_SO_4_ ⋅ H_2_O in combination with the use of carbon‐coated Al foil as current collector on the long‐term stability of NCM811||graphite full‐cells can be seen in Figure 5. Table S2 shows the initial C_Eff_, initial discharge capacities at 0.1 and 1 C after four formation cycles (5th cycle), and the state‐of‐health (SOH) after 400 cycles for all cells based on the discharge capacity of the 5th cycle. The full‐cells using the reference NMP‐processed cathode exhibit a capacity of 172 mAh g^−1^ at 1 C and reached 84 % SOH after 400 cycles. Due to the degradation of the cathode active material and the Li leached out of the layered structure, the aqueously processed electrode without Li_2_SO_4_ ⋅ H_2_O and with standard Al foil as current collector reaches the 80 % SOH after only around 200 cycles. By changing the current collector to carbon‐coated Al foil, the discharge capacity is slightly lower, but the capacity retention is clearly improved (83 % SOH after 400 cycles), which is probably related to improved electronic conductivities by using carbon‐coated Al foil (Table 2). In contrast, the discharge capacity as well as the capacity retention are improved for cells containing aqueously processed electrodes with Li_2_SO_4_ ⋅ H_2_O and bare Al foil as current collector (Table S2). However, it is observed that there is an optimum amount for the added Li_2_SO_4_ concentration to the electrode paste at which discharge capacity and cycling stability reach a maximum. The positive impact of Li_2_SO_4_ ⋅ H_2_O is clearly visible by using a concentration of 2 wt %. For higher concentrations of additive (5 wt %), the higher resistance of the electrodes surpasses the positive influence of Li_2_SO_4_ ⋅ H_2_O. By combining the carbon‐coated Al foil with 2 wt % of Li_2_SO_4_ ⋅ H_2_O, the discharge capacity as well as the capacity retention is similar or even better than for cells using the NMP‐processed cathodes. These results give evidence that the aqueous processing of Ni‐rich layered oxides with the appropriate additive concentration and the use of carbon‐coated Al current collector to prevent corrosion is a feasible strategy to enable high‐energy densities and long lifetime.

**Figure 6:**
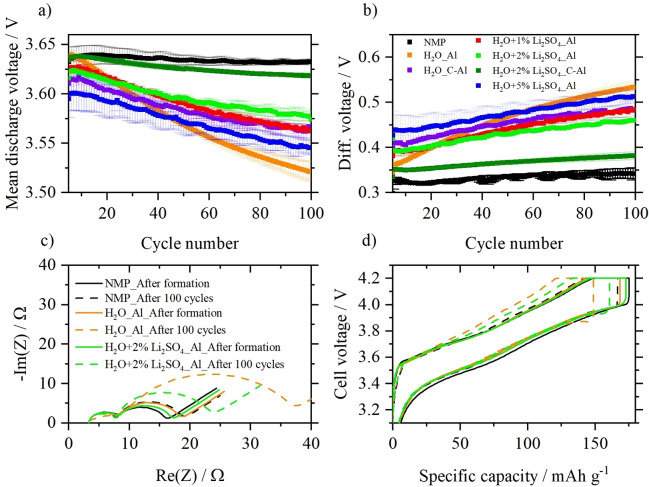
Detailed electrochemical characterization of the cathodes prepared by different processing methods in NCM811||graphite full cells at a rate of 1 C. (a) Evolution of mean discharge voltages during the first 100 cycles. (b) Difference between mean charge/discharge voltage (Δ*V*) during the first 100 cycles. (c) Fitted impedance spectra of NCM811||graphite full cells charged to 3.9 V after formation (solid lines) and after 100 cycles (dashed lines). (d) Representative cell voltage profiles after formation and after 100 cycles. Cell voltage range: 2.8–4.2 V, N/P ratio: 1.15 : 1.00, electrolyte: 1 m LiPF_6_ in 3 : 7 vol % EC/EMC+2 wt % VC.

The average cell discharge voltage (*U*_dis_) as well as the difference between charge and discharge voltage (Δ*V*) are compared in Figure 6a,b to gain a better understanding of the improved electrochemical performance after addition of Li_2_SO_4_ ⋅ H_2_O to the electrode paste. Both parameters can give an indication of the evolution of internal resistances and polarization growth during cycling. A lower discharge voltage might indicate a higher impedance for Li^+^ insertion from the cathode and/or a higher impedance for the Li^+^ extraction from the anode.^[26]^ Furthermore, a lower mean cell voltage will directly result in lower specific energies (Figure S9) and energy densities, which are key parameters of relevance for the practical application of Ni‐rich cathode materials in high‐energy LIBs. As can be seen, the mean discharge voltage of cells containing cathodes processed with 5 wt % Li_2_SO_4_ ⋅ H_2_O is around 30 mV lower than those of other electrodes, which is probably due to the higher resistance because of the presence of high amounts of additive. Cells with cathodes processed with water and coated on bare Al foil have a high initial discharge voltage of around 3.63 V, which is comparable to the NMP reference cells However, the discharge voltage decreases much faster compared to electrodes with Li_2_SO_4_ ⋅ H_2_O or carbon‐coated Al foil, which also correlates with the poor cycling stability (Figure 5). This severe voltage fade might indicate a pronounced formation of internal resistances (e. g., surface layers due to exposure of the active material to water) instead of the influence of the electrode resistance (Table 2). It is worth mentioning that the combination of carbon‐coated Al foil with 2 wt % of Li_2_SO_4_ ⋅ H_2_O leads to high discharge voltages with only a minimal decrease (≈15 mV) during 100 cycles. The same trend is observed in the evolution of Δ*V*, as shown Figure 6b. NCM811||graphite cells using cathodes with 2 wt % Li_2_SO_4_ show good capacity retention as well as low resistance growth upon cycling, which leads to high initial specific energies above 550 Wh kg^−1^ in the 5th cycle as well as high energy efficiencies above 90 % (Figure S9), which is another critical parameter for practical application.^[35]^

Cells were further investigated by electrochemical impedance spectroscopy (EIS) to evaluate the impact of the different processing conditions on the resistance of the cells to get a better understanding of the long‐term cycling (Figure 6c). Prior to the measurement, the cells after the formation cycles and 100 cycles were charged to 50 % state of charge (SOC) to reduce the influence of fully charged/discharged electrodes. All measurements were performed for cells containing cathodes coated on pure Al foil as current collector. The Nyquist plots show inductive behavior at high frequencies followed by two semi‐circles and a straight line at lower frequencies. According to literature,^[36]^ the inductive part describes the resistance *R*1 of the cell‐setup and the electrolyte. The first semi‐circle describes the two‐dimensional resistance of electrode interfaces (*R*_EI‐2D_), for example, electrode/current collector, electrode/electrolyte, and the resistance of three‐dimensional electrode interphases (*R*_EI‐3D_), for example, solid electrolyte interphase (SEI) at the anode or cathode electrolyte interphase (CEI) at the cathode.[^36^, ^37^, ^38^] The contribution of both interphase‐caused resistances is abbreviated in the following discussion with *R*_EI_. The second semi‐circle corresponds to resistances, which are generated from charge‐transfer (*R*_CT_) reactions, for example, by Li^+^ (de‐)intercalation. The straight line (Warburg impedance) at the low‐frequency range with a constant slope describes the solid‐state diffusion of Li^+^. It is very important to mention that both the anode and the cathode contribute to the formation of the cell impedance, and with the use of a two‐electrode set‐up it is difficult to distinguish the contributions from the individual electrodes.^[36]^ However, here we assume that changes during cycling should result from the contributions of the differently processed cathodes, as the other components (graphite anode, separator, and electrolyte) did not change. Figure S10 shows the equivalent circuit used to fit the EIS data results and resistances over cycling, respectively. The *R*_EI_ is represented as a parallel connection of the resistance *R*2 and the capacitance from the constant phase element CPE1, while *R*_CT_ is simulated as a parallel connection of *R*3 with CPE2 (Figure S10).^[39]^ The *R*_EI_ for all cells has a value of around 8 Ω and is independent of the number of cycles and the processing method. For the NMP‐processed electrode, the *R*_CT_ has a value of 16  Ω, which shifts to 19 Ω after 100 cycles, probably due to ageing effects of the NCM811. The aqueously processed electrodes have larger *R*_CT_ values after formation. Interestingly, the addition of 2 wt % Li_2_SO_4_ ⋅ H_2_O leads to a lower *R*_CT_ value of 17 Ω compared to 19 Ω without additive. After 100 cycles, the positive impact of Li_2_SO_4_ ⋅ H_2_O in cell impedance is evident. Electrodes with Li_2_SO_4_ ⋅ H_2_O have a charge‐transfer resistance of 25 Ω compared to 37 Ω for the electrodes without additive. Therefore, the addition of Li_2_SO_4_ ⋅ H_2_O has a severe impact on the charge‐transfer mechanism in aqueously processed electrodes. This observation is also confirmed by the cell voltage profiles after formation and after 100 cycles (Figure 6d). These profiles are similar for the three electrodes after formation (NMP‐processed and aqueously processed with/without Li_2_SO_4_), with slightly larger capacities for the NMP and Li_2_SO_4_ ⋅ H_2_O containing electrodes, respectively. After 100 cycles, the charge and discharge cell voltage profiles of the aqueously processed electrodes differ. Charging of the electrodes is accompanied by larger over‐voltages, which indicates hindered Li^+^ extraction from the cathode. The over‐voltage of electrodes processed with Li_2_SO_4_ ⋅ H_2_O during charge is lower than the over‐voltage without Li_2_SO_4_ ⋅ H_2_O. On the one hand, the exposure of the cathode active material to water likely leads to leaching out of Li and the formation of a surface film as seen by SEM and XPS analyses, which can suppress the charge‐transfer reaction and hinder the Li extraction from the cathode. On the other hand, the Li_2_SO_4_ ⋅ H_2_O may form a surface film on the cathode particles, which eases Li^+^ extraction by decreasing the charge‐transfer resistance as reported in literature.^[40]^ It may also be possible, that both effects are responsible for the better impedance value. Storage experiments of Li_2_SO_4_ ⋅ H_2_O combined with ion‐chromatography measurements (see experimental details) give evidence that Li_2_SO_4_ ⋅ H_2_O is not soluble in the used electrolyte. Therefore, the positive impact of Li_2_SO_4_ ⋅ H_2_O stems probably from the surface coating rather than positive influences on the anode (e. g., as pre‐lithiation additive that provides excess active Li to compensate for active lithium losses during SEI formation).

## Conclusions

In this work, the impact of Li_2_SO_4_ ⋅ H_2_O as processing additive for the aqueous manufacturing route of NCM811 cathodes on the material properties, electrode properties and electrochemical performance was systematically evaluated. The addition of Li_2_SO_4_ ⋅ H_2_O during high‐energy dispersion of NCM811 in water hindered an increase of the specific surface area. Thermogravimetric analysis (TGA), scanning electron microscopy (SEM), and X‐ray photoelectron spectroscopy (XPS) experiments gave evidence that the Li_2_SO_4_ additive influences the surface composition and morphology of aqueously‐processed NCM811 materials, leading to formation of a protective coating on the surface of NCM811 particles, which subsequently stabilizes the NCM811 against degradation.

The electrode and electrochemical properties were also influenced by the processing method. By using carbon‐coated Al foil, the corrosion of the current collector was remarkably suppressed, leading to an electrode surface morphology without significant cracks, similar to the *N*‐methyl‐2‐pyrrolidone (NMP)‐processed electrodes. Aqueously processed electrodes exhibit a higher adhesion between current collector and electrode as well as low through‐plane resistances. The addition of 2 wt % Li_2_SO_4_ ⋅ H_2_O increased the adhesion, but also the resistance of the electrodes. The electrochemical performance was evaluated for the different processing methods in NCM811||Li metal cells and NCM811||graphite cells. Specific discharge capacities were gradually increased with the amount of Li_2_SO_4_ ⋅ H_2_O (1–5 wt %) added. Electrodes without Li_2_SO_4_ ⋅ H_2_O on pure Al foil reached 80 % state of health after only 200 cycles, while the use of carbon‐coated Al foil extended the cycle life to 400 cycles. The addition of small amounts of Li_2_SO_4_ ⋅ H_2_O also improved the capacity retention. A concentration of 2 wt % Li_2_SO_4_ ⋅ H_2_O during cathode paste processing shows the best trade‐off between high specific capacities and long cycle life and even outperforms NMP‐processed electrodes. Impedance measurements of electrodes with and without Li_2_SO_4_ ⋅ H_2_O confirmed that the growth of the charge‐transfer resistance during cycling is significantly higher for aqueously processed electrodes without Li_2_SO_4_ ⋅ H_2_O.

The use of Li_2_SO_4_ ⋅ H_2_O as additive for the processing of Ni‐rich layered oxide electrodes might be an effective strategy to overcome some of the challenges of aqueous‐processing routes. With this approach, it might be possible to establish the aqueous processing of Ni‐rich layered oxides in larger scales, leading to a low‐cost and more environmentally friendly battery production.

## Experimental Section

### Characterization of cathode materials and Li_2_SO_4_ ⋅ H_2_O

5 g of Ni‐rich layered oxide cathode active material [LiNi_0.8_Co_0.1_Mn_0.1_O_2_ (NCM811), *D*_90_≤19.1 μm T81RX, Shanshan] was dispersed (Dispermat LC30, VMAGetzmann GmbH) at a speed of 15000 rpm for 1 h in either 5 mL of NMP (anhydrous, purity: 99.5 %, Sigma‐Aldrich), 5 mL deionized water, or 5 mL deionized water with 2 wt % Li_2_SO_4_ ⋅ H_2_O (purity: 99 %, Sigma‐Aldrich), respectively, to evaluate possible changes of the NCM811 surface morphology and crystallinity upon contact with the processing solvents. To quantify the lithium loss of the CAMs, the dispersion was either filtrated (F) with a folded filter (Rotilabo, type 601P, Carl Roth*)* or not filtrated (NF). The powder (NF) was dried at 80 °C, hand‐ground with mortar and pestle, and stored in a dry room until further use (dew point ≤−50 °C, relative humidity of 0.16 %).

The surface morphology of the pristine and solvent‐treated NCM811 materials was analyzed via SEM using a Carl Zeiss AURIGA field emission microscope with an acceleration voltage of 3 kV and a working distance of 4 mm. EDX mapping was performed with an acceleration voltage of 20 kV and a working distance of 5 mm using the software INCA. The stoichiometry was determined using ICP‐OES (Spectro ARCOS EOP) with an axial positioned plasma torch. Measurement conditions were applied according to Evertz et al.^[41]^ For the analysis of the specific surface area, the powders were dried at 200 °C and adsorbed water was removed overnight under reduced pressure with a VacPrep 061 (Micromeritics GmbH). Afterwards, the specific surface area was determined with BET calculation using Krypton adsorption on an ASAP2020 (Micromeritics GmbH*)*. Powder XRD (Bruker D8 Advance) was performed between 10–90° at a step size of 0.02° s^−1^ using Cu‐K_α_ radiation (*λ*=0.154 nm) at 40 kV and 20 mA with a divergence slit of 0.6 mm. The diffraction patterns (see Figure S1) were Rietveld‐refined based on a hexagonal α‐NaFeO_2_ structure with a space group *R*‐3 *m* using Topas Academic V6 (Bruker AXS GmbH). For the refinements, Li was assumed to occupy 3*a* sites, transition metals (TMs) were assumed to occupy 3*b* sites, and oxygen was assumed to occupy 6*c* sites. The occupation of Ni^2+^ in the lithium layer was also quantified to account for the Li/Ni cation mixing disorder.

TGA of the dried cathodes and NCM811 was performed on a TGA Q5000‐IR (TA Instruments) to evaluate their thermal stability. Samples were heated under helium flow between 30 and 900 °C with a heating ramp of 10 K min^−1^. The decomposition products were identified using a coupled mass spectrometer ThermoStar GSD 301 T3 (Pfeiffer Vacuum).

XPS measurements were conducted using a JEOL JPS‐9030 setup, employing an achromatic Mg source with 300 W power for excitation. A hemispherical analyzer with pass energy of 50 eV (surveys) and 30 eV (narrow scans) was used to detect the emitted photoelectrons. The binding energy scale was calibrated by measuring a sputter cleaned gold foil just before the measurements and setting the Au 4 f peak to 84.00 eV. No signs of charging were observed during the measurements. The NMC powders were pressed into pellets for the XPS measurements. All samples were exposed to air for the same amount of time before introduction into the vacuum setup.

Solubility experiments were performed by storing 0.02 g Li_2_SO_4_ ⋅ H_2_O for 1 week in 1 mL 1 m LiPF_6_ in 3 : 7 vol % ethylene carbonate (EC)/ethyl methyl carbonate (EMC; Solvionic; purity: battery grade). Afterwards, the dispersion was filtrated and analyzed with Ion‐chromatography (IC). IC measurements were performed on an 850 Professional IC (Metrohm, Herisau, Switzerland) with conductivity detection (CD) A Metrosep A Supp 4‐ (250×4.0 mm, 9 μm; Metrohm) with a Metrosep A Supp 4/5 guard column was used for isocratic anion separation at 40 °C, and a flow rate of 1 mL min^−1^ was applied. The developed method is based on a report by Kraft et al.,^[42]^ and further parameters and sample preparation were applied according to a report by Henschel et al.^[43]^

### Electrode preparation and characterization

For the aqueous processing of the cathode paste, sodium‐carboxymethyl cellulose (Na‐CMC, Walocel CRT 2000 PPA12, Dow Wolff Cellulosics, 1.5 wt %) was first dissolved overnight in deionized water. Afterwards, the conductive carbon (Super C65, Imerys Graphite & Carbon, 3 wt %), Li_2_SO_4_ ⋅ H_2_O (*x* wt %), and the NCM811 active material (94−*x* wt %) were added and the paste was homogenized by a high‐energy disperser (Dissolver Dispermat LC30, VMAGetzmann GmbH) for 1 h at 15000 rpm. Finally, the ENEOS acrylate binder (ENEOS Corporation, 1.5 wt %) was added to the electrode suspension and the rotational speed of the disperser was decreased to 500 rpm during the last 5 min. The amount of Li_2_SO_4_ within the electrode paste ranged between 1 and 5 wt %, depending on the solid content (≈50 %) of the electrode dispersion.

The reference NCM811 cathode was prepared by using the conventional electrode processing route using fluorinated binders and organic processing solvent. The Ni‐rich positive electrodes consisted of 94 wt % NCM811 (D_90_≤19.1 μm T81RX, Shanshan) as active material, 3 wt % conductive carbon (Super C65, Imerys Graphite & Carbon, 3 wt %), and 3 wt % PVdF as binder (Solef 5130, Solvay) dissolved in NMP (anhydrous, purity: 99.5 %, Sigma‐Aldrich) with a solid content of 50 %. The pH value of the aqueous electrode dispersions was measured after dispersion with the Dispermat for 60 min (before coating) with a pH tester with two‐point calibration (VWR, pH 20 series).

The prepared electrode pastes were coated with a doctor‐blade (Zehntner GmbH) and an automatic film applicator (Sheen Instruments) on Al foil (cleaned with ethanol, 20 μm, Nippon foil) and carbon‐coated Al foil (20 μm, Nippon, UACJ Foil Corporation). The average active mass loading of the cathode was around 5 mg cm^−2^ for investigations in NCM811||Li metal cells and around 12 mg cm^−2^ for NCM811||graphite cells. The cathodes were dried at 80 °C for 2 h, punched out with a diameter of 14 mm, and dried in a Büchi B‐585 glass drying oven under reduced pressure (<50 mbar) at 120 °C for 12 h. The electrodes were pressed with a hydraulic hand‐press (Graseby Specac press) to reach a porosity of around 40 %.

The negative electrodes (anodes) were prepared by mixing 95 wt % commercial synthetic graphite (SMG−A5, Hitachi Chem), 1.5 wt % styrene‐butadiene‐rubber (SBR, SB5521, LIPATON; Polymer Latex GmbH), and 3.0 wt % sodium‐carboxymethyl cellulose (Na‐CMC, Walocel CRT 2000 PPA12, Dow Wolff Cellulosics) as binders and 0.5 wt % carbon black (Super C65, Imerys Graphite & Carbon) as conductive agent in deionized water. The anode paste was coated onto copper foil (10 μm, Nippon foil), dried and calendared to reach 30 % porosity. Anode disks with a diameter of 15 mm and an average active mass loading of around 7 mg cm^−2^ were punched out and dried in a Büchi B‐585 glass drying oven under reduced pressure (<50 mbar) at 120 °C for 12 h.

The adhesion force as well as the through plane electronic conductivity were measured for the whole cathode sheets on a Zwick Roell testing machine (Zwick Roell, testControl II, 2.5kN). For the measurement of the adhesion force, the electrode sheet was fixed with double‐sided sticky tape on a steel sample holder (surface area 6.45 cm^2^ per measuring point with 5 measuring points per sample holder). A second sample holder was prepared with double‐sided sticky tape and pressed for 60 s with 2000 N onto the cathode. Afterwards, the sample holders were separated with a peel‐off speed of 10 mm min^−1^. For the electronic conductivity measurement, the cathode sheet was placed between two copper electrodes. The copper electrodes had an initial working distance of 2 mm. During the measurement, the distance between the copper electrodes was decreased by adjusting the force. The through‐plane conductivity or rather the resistance was monitored with a Resistomat 2316 from Burster. For better comparison, the resistance at the end of the measurement (at 100 N) was used.

### Cell assembly and electrochemical characterization

All electrochemical investigations were performed in a two‐electrode configuration^[33]^ in coin cells (CR2032, Hohsen) with a polymer membrane (1‐layer, 16 mm Ø, Celgard 2500, Celgard) as separator. The separator was soaked with 35 μL of electrolyte, that is, 1 m LiPF_6_ in 3 : 7 vol % EC/EMC (Solvionic; purity: battery grade) with 2 wt % vinylene carbonate (VC, Solvionic, purity: battery grade). For NCM811||Li metal cells, the prepared cathode (1 mAh cm^−2^) was paired with Li metal foil (Ø15 mm, 500 μm in thickness; purity: battery grade, China Energy Lithium). For NCM811||graphite cells, the cathode (2.2 mAh cm^−2^) was matched with a graphite anode (2.5 mAh cm^−2^), leading to negative/positive capacity balancing ratio of N/P 1.15 : 1.00 (based on the 2nd cycle discharge capacity in NCM811||Li metal cells and graphite||Li metal cells). The coin cells were assembled in a dry room atmosphere and the reproducibility of the electrochemical data was verified by assembling at least three cells for each sample.

Electrochemical evaluation was performed via constant current (CC) charge–discharge cycling on a Maccor Series 4000 battery tester (Maccor, Inc.) at 20 °C. The rate capability as well as the stability at higher cut‐off voltages was first investigated in NCM811||Li metal cells. A specific current of 190 mA g^−1^ (2.9–4.3 V) was defined as 1 C. First, the cells were rested at open‐circuit voltage (OCV) for 6 h to allow the wetting of the electrodes and separator. Afterwards, the cells were cycled for two formation cycles at 0.1 C, three cycles at 0.2 C, and five cycles each at 0.33, 0.5, 1, and 3 C. To prevent Li metal plating on the anode, the cells were always charged at a constant rate of 0.2 C while the discharge rate ranged between 0.1 and 3 C. The cell voltage window was set to 2.9 and 4.3 V. After the rate capability investigations, the NCM811||Li metal cells were cycled for two cycles at 0.1 C followed by 15 cycles each at 0.33 C up to 4.3, 4.4, and 4.5 V as upper cut‐off voltages.

The long‐term cycling stability was evaluated in NCM811||graphite full‐cells in a cell voltage window between 2.8 and 4.2 V. Full‐cells were charged to 1.5 V for 15 min prior to 6 h at OCV to prevent Cu current collector dissolution. Cells were then cycled for four cycles at 0.1 C to allow formation of the SEI. Afterwards, the cells were cycled at 1 C for 400 cycles with two regeneration cycles at 0.1 C each 100 cycles (1 C=190–196 mA g^−1^ based on the 2nd cycle discharge capacity in NCM811||Li metal cells). After each charging step, a constant voltage (CV) step was performed with the limiting conditions of either achieving a time limit (≥30 min) or a specific current limit (≤0.05 C).

Electrochemical impedance spectroscopy (EIS) was performed using a VMP potentiostat (Bio‐logic) between 100 kHz and 10 mHz with an amplitude of 10 mV. Prior to each measurement, the cells were charged to 50 % state‐of‐charge. The data was fitted (based on the equivalent circuit shown in Figure S10) in the software EC‐Lab (Bio‐logic).

## Conflict of interest

The authors declare no conflict of interest.

1

## Supporting information

As a service to our authors and readers, this journal provides supporting information supplied by the authors. Such materials are peer reviewed and may be re‐organized for online delivery, but are not copy‐edited or typeset. Technical support issues arising from supporting information (other than missing files) should be addressed to the authors.

Supporting Information

## Data Availability

Research data are not shared.
