# Supplementary material for: Enabling Aqueous Processing of Ni‐Rich Layered Oxide Cathode Materials by Addition of Lithium Sulfate
Source: ChemSusChem. 2022 Dec 14;16(2):e202202161. doi: 10.1002/cssc.202202161 (PMC10107986; doi:10.1002/cssc.202202161)
Supplement: Supplementary file 1 — Supporting Information [file CSSC-16-0-s001.pdf]

ChemSusChem

Supporting Information

Enabling Aqueous Processing of Ni-Rich Layered Oxide Cathode Materials by Addition of Lithium Sulfate

Marcel Heidbüchel, Thorsten Schultz, Tobias Placke, Martin Winter, Norbert Koch, Richard Schmuch,* and Aurora Gomez-Martin*© 2022 The Authors. ChemSusChem published by Wiley-VCH GmbH. This is an open access article under the terms of the Creative Commons Attribution License, which permits use, distribution and reproduction in any medium, provided the original work is properly cited.

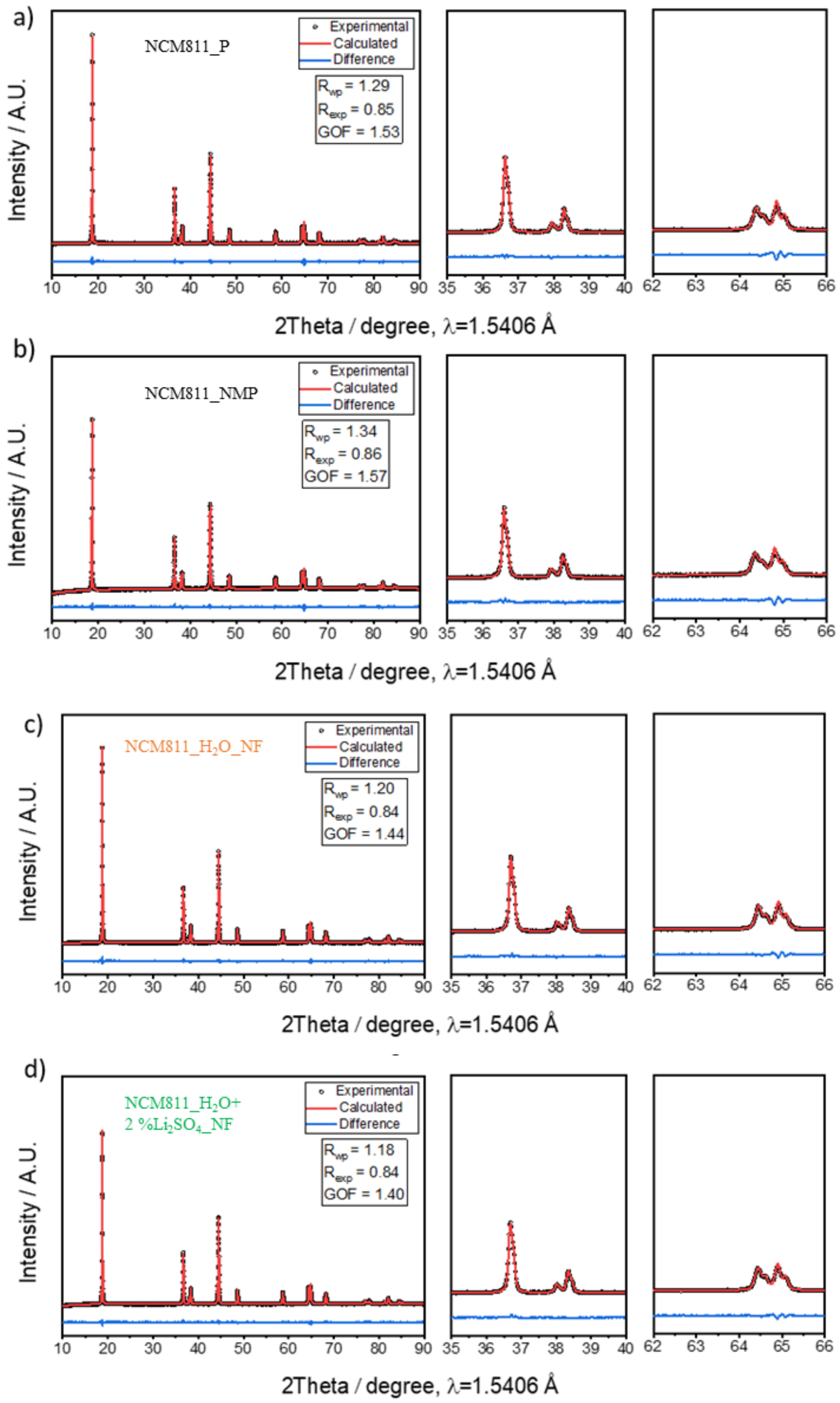

Figure S1: Detailed Rietveld refinements of XRD patterns of NCM811 powders after different processing methods: (a) pristine NCM811, (b) NCM811 treated in NMP for 1h (c) NCM811 treated in H_2O for 1h (not filtrated; NF) and (d) NCM811 treated in H_2O + 2 wt.% Li_2SO_4 for 1h (not filtrated; NF). The black empty dots show the original data points, the solid red lines the calculated results, while the blue lines show the differences between the measured and calculated data. R-weighted pattern (R_{wp}), R-expected (R_{exp}) and goodness of fit (GOF) are shown in the inset.

Table S1: Rietveld refinement results for XRD powder analyses of pristine NCM811 and NCM811 after contact with different solvents.: R-weighted pattern (R_{wp}), R-expected (R_{exp}), goodness of fit (GOF), lattice parameters *a* and *c*, unit cell volume (V), Li/Ni mixing.

Material	NCM811_P	NCM811_NMP	NCM811_H_2O_NF	NCM811_H_2O+2 % Li_2SO_4_NF
R_{wp}	1.29	1.34	1.20	1.18
R_{exp}	0.85	0.86	0.84	0.84
GOF	1.53	1.57	1.44	1.40
a /10^{-10}m	2.8732(2)	2.8741(2)	2.8731(2)	2.8732(2)
c /10^{-10}m	14.213(2)	14.214(2)	14.211(2)	14.211(2)
V / 10^{-30}m^3	101.61(2)	101.69(2)	101.59(2)	101.60(2)
Li / Ni mixing/ %	2.30(1)	3.40(1)	2.46(7)	2.58(1)

Thermogravimetric analysis coupled with mass spectrometry was performed to investigate the thermal stability of the CAMs with or without water exposure, which provides insights into the amount of surface species and the stability of the different binder systems (**Figure S2**). The temperature-dependent mass signals m/z =18, 28, 32 and 44 are depicted for all CAMs in **Figure S2c-f** and can be assigned to the release of H_2O, CO, O_2, and CO_2 species, respectively. As can be seen in **Figure S2a,b**, the processing method of these materials also strongly influences the thermogravimetric behavior. Overall, the mass loss of the aqueously-processed CAMs upon heating is clearly higher than the mass loss for the pristine NCM811. The formed surface residues after contact with water, *e.g.*, bicarbonates, carbonates and hydroxides can also thermally decompose accompanied by the release of H_2O and CO_2.[1],[2] Sicklinger *et al.* suggested the formation of transition metal carbonates / hydroxides instead of Li-containing residues, which are responsible for CO_2 release at ≈350 °C. [1] According to these results, the mass loss of NCM811 CAMs with different processing methods indicates thermal decomposition of surface residues. The release of water below 150 °C might be due to the dehydration of $Li_2SO_4 \cdot H_2O$ followed by a release of chemisorbed water from NCM811 (**Figure S2c**). Above 150 °C, the H_2O and O_2 release could indicate thermal decomposition of Ni oxyhydroxide species together with the formation of NiO.[3] The

release of CO at 200 °C and CO_2 below 500 °C indicates thermal decomposition of surface species, *e.g.* carbonates from Li_2SO_4-treated sample. In addition, the 'NCM811_H_2O' sample releases only CO_2 above 600 °C indicating the formation of thermally more stable carbonates on its surface, which differ from the carbonates of the Li_2SO_4-treated sample (**Figure S2d, f**). Furthermore, different thermal decomposition of surface residues compared to 'NCM811_P' or 'NCM_H_2O' samples confirm the assumption that Li_2SO_4 influences the NCM811 surface during aqueous processing.

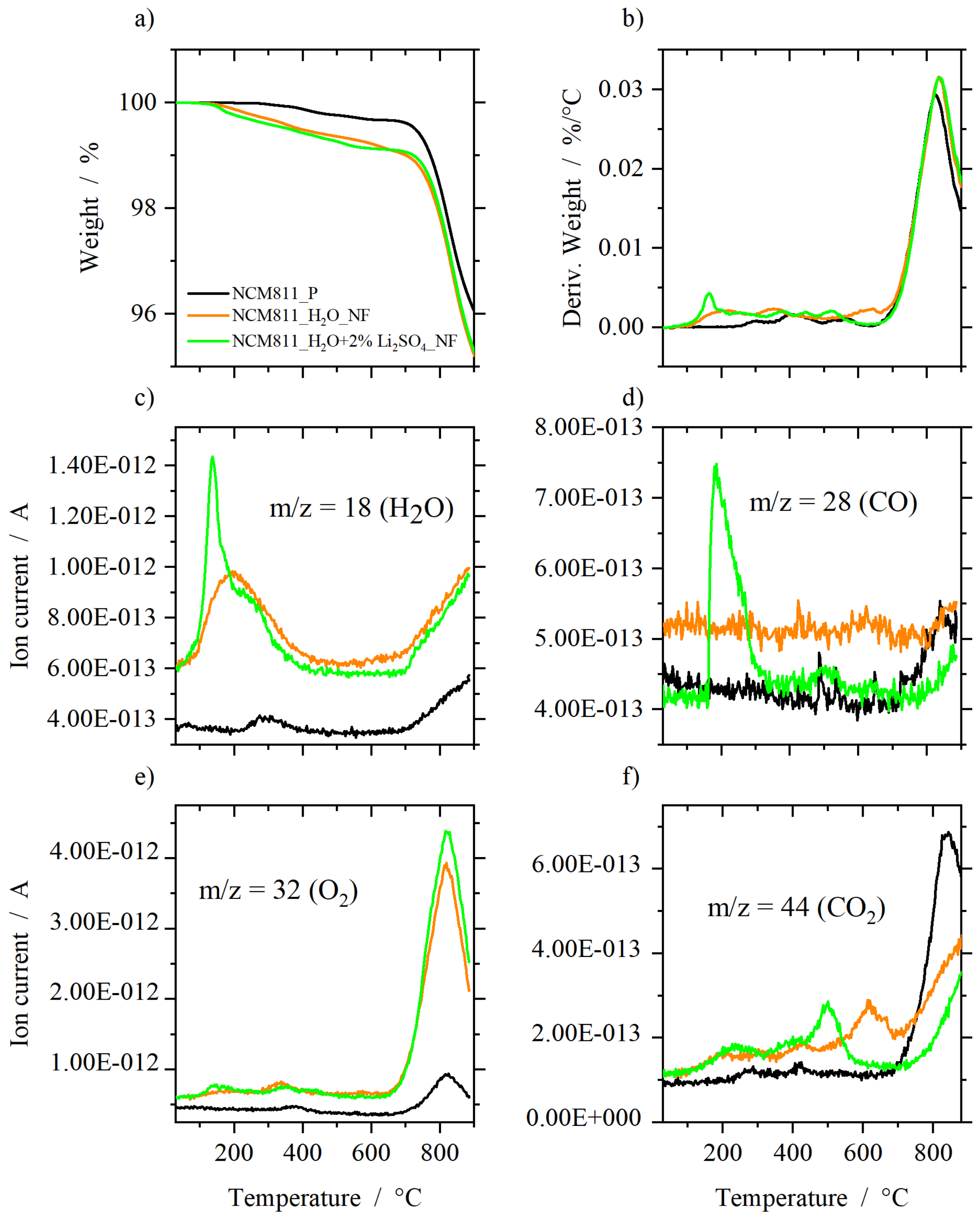

Figure S2: Thermogravimetric analysis coupled with mass spectrometry results showing the temperature-dependent behavior of NCM811 CAMs powders with different processing methods. (a) Weight loss and (b) derivative weight loss of the electrodes. Mass spectra of (c) H_2O, (d) CO, (e) O_2, (f) CO_2.

In addition, the onset temperature of the mass loss for the aqueously-processed electrodes is at ≈200 °C, while the onset for NMP-processed electrodes is at ≈ 400 °C. The mass loss for the NMP-processed electrodes has two maxima at ≈450 °C and ≈550 °C, which are related to the thermal decomposition of the PVdF binder with associated H_2O and

CO_2 release (**Figure S3c** and **d**). The mass loss curve of the aqueously-processed electrodes shows four maxima at ≈250 °C, ≈350 °C, ≈470 °C and ≈525 °C, which can be attributed to the thermal decomposition or decarbonization of CMC and the acrylate binders.[4, 5] The first maximum of the mass loss is related to thermal decomposition of CMC with the release of structurally bonded H_2O (**Figure S3c**).[5, 6] Ongoing with the release of H_2O, the decomposition of CMC results in CO and CO_2 release at 350 °C (**Figure S3d and e**).[4] The last two maxima at ≈470 °C and ≈525 °C most likely stem from the thermal decomposition of the acrylate binder, leading to a release of H_2O and CO_2 (**Figure S3c and f**).

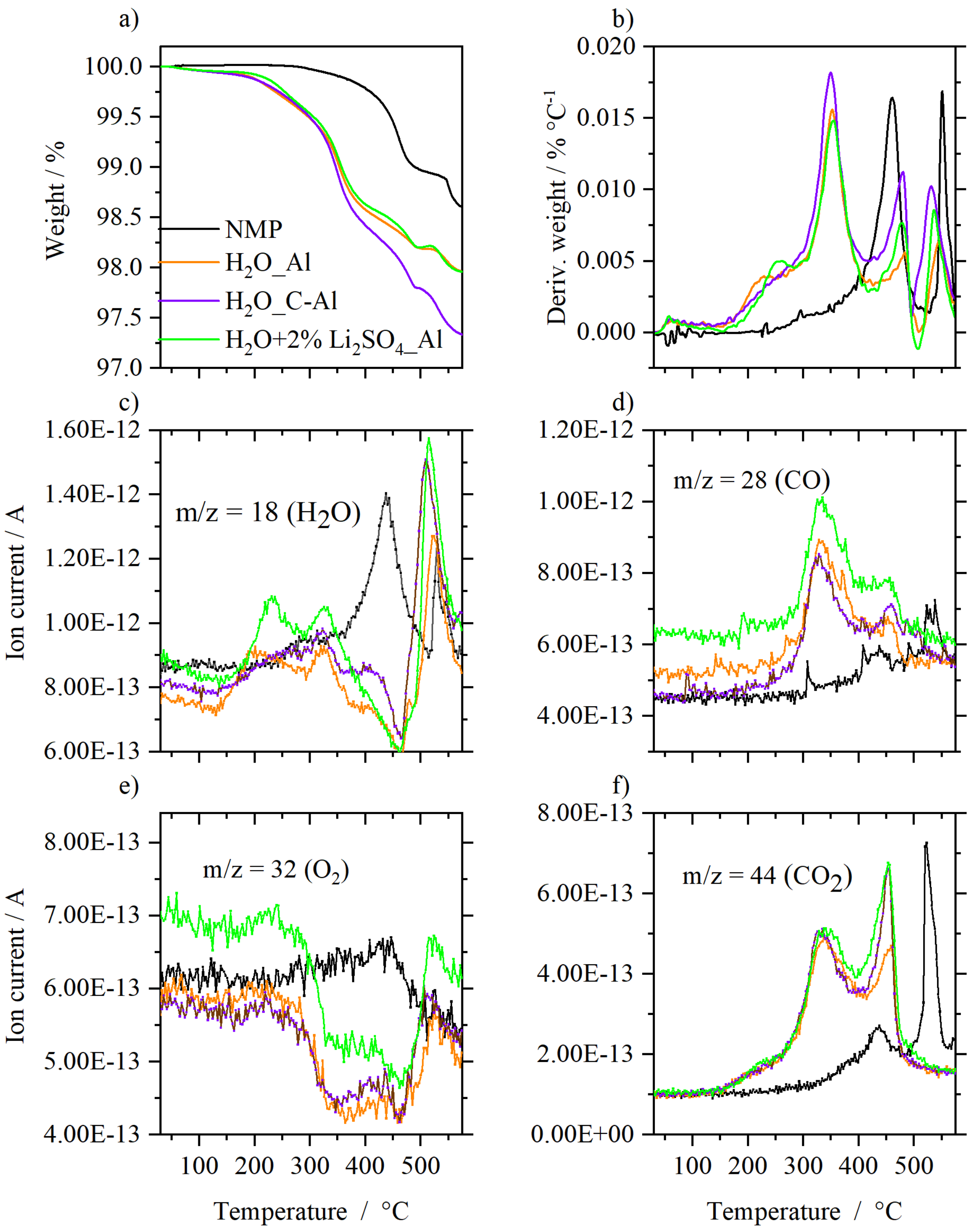

Figure S3: Thermogravimetric analysis coupled with mass spectrometry results showing the temperature-dependent behavior of NCM811 cathode electrodes with different processing methods. (a) Weight loss and (b) derivative weight loss of the electrodes. Mass spectra of (c) H2O, (d) CO, (e) O2, (f) CO2.

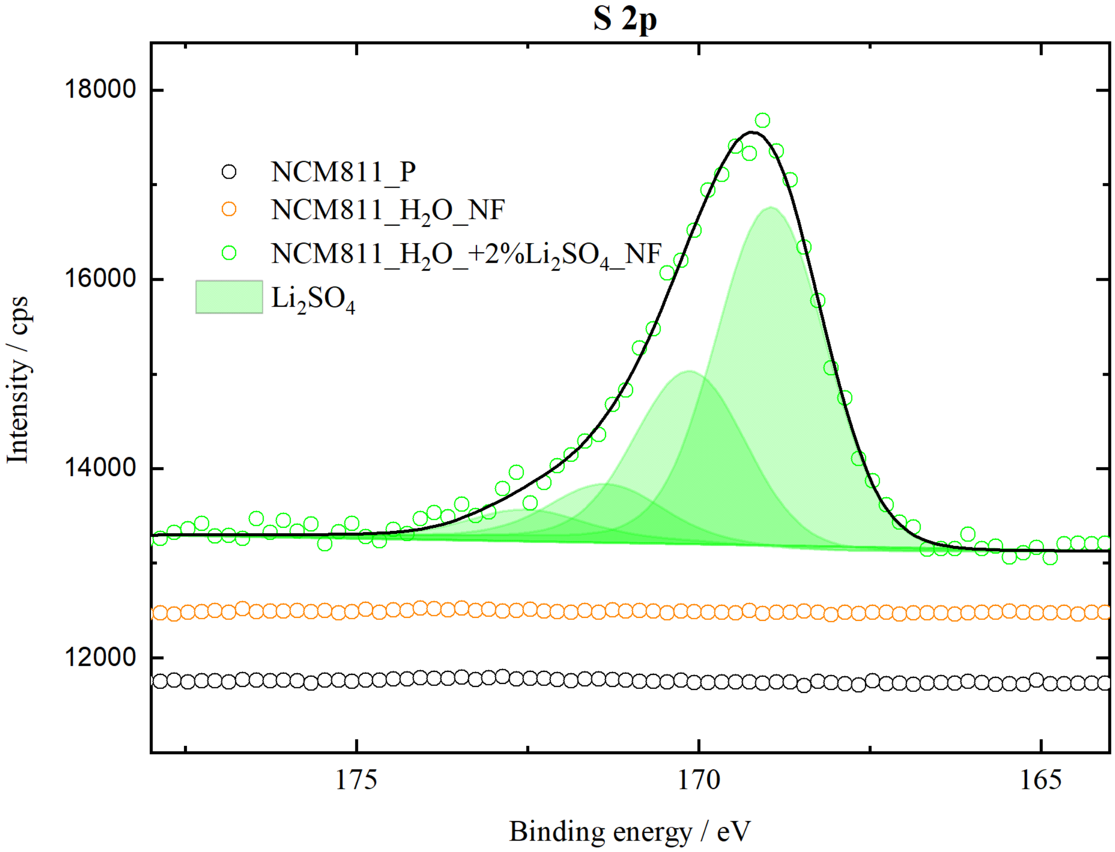

Figure S4: XPS core level spectra of the S 2p peak of the NCM 811 electrodes processed with different processing conditions.

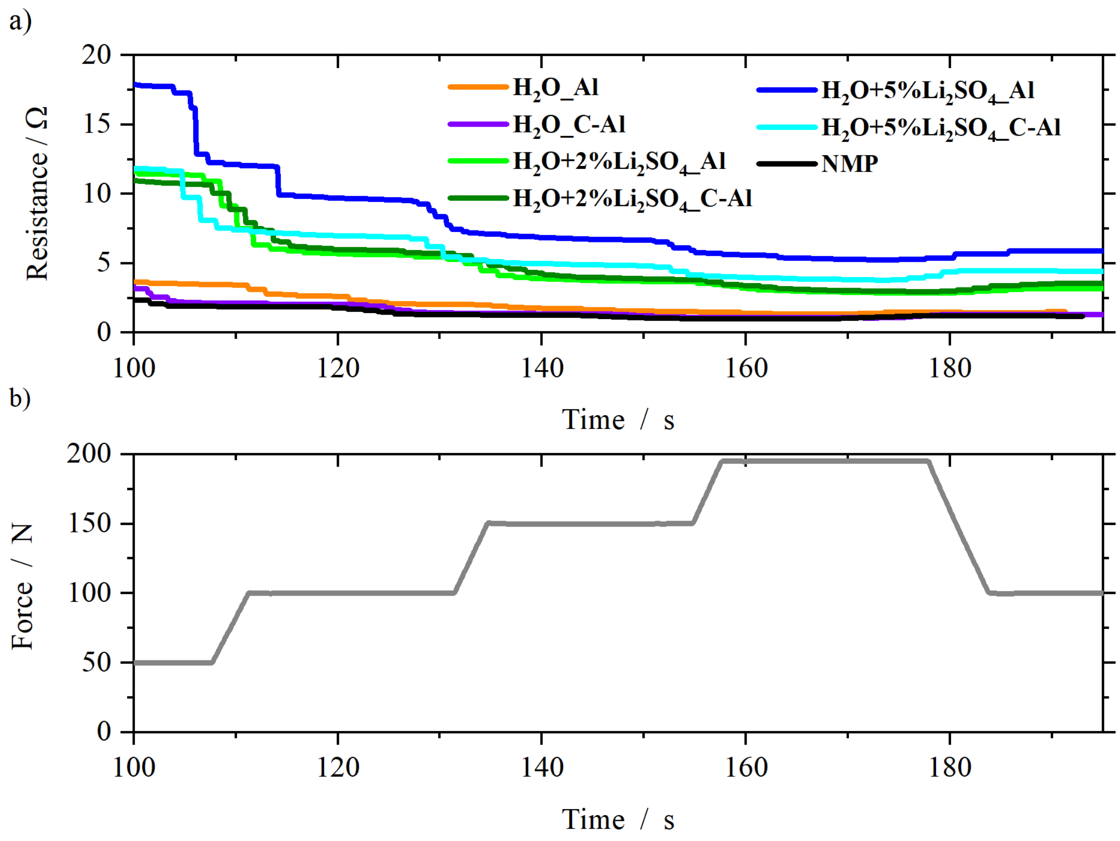

Figure S5: Through-plane electronic conductivity measurements of the pristine NCM811 electrodes without calendaring. Time-dependent profile of a) resistance and b) force during the measurement.

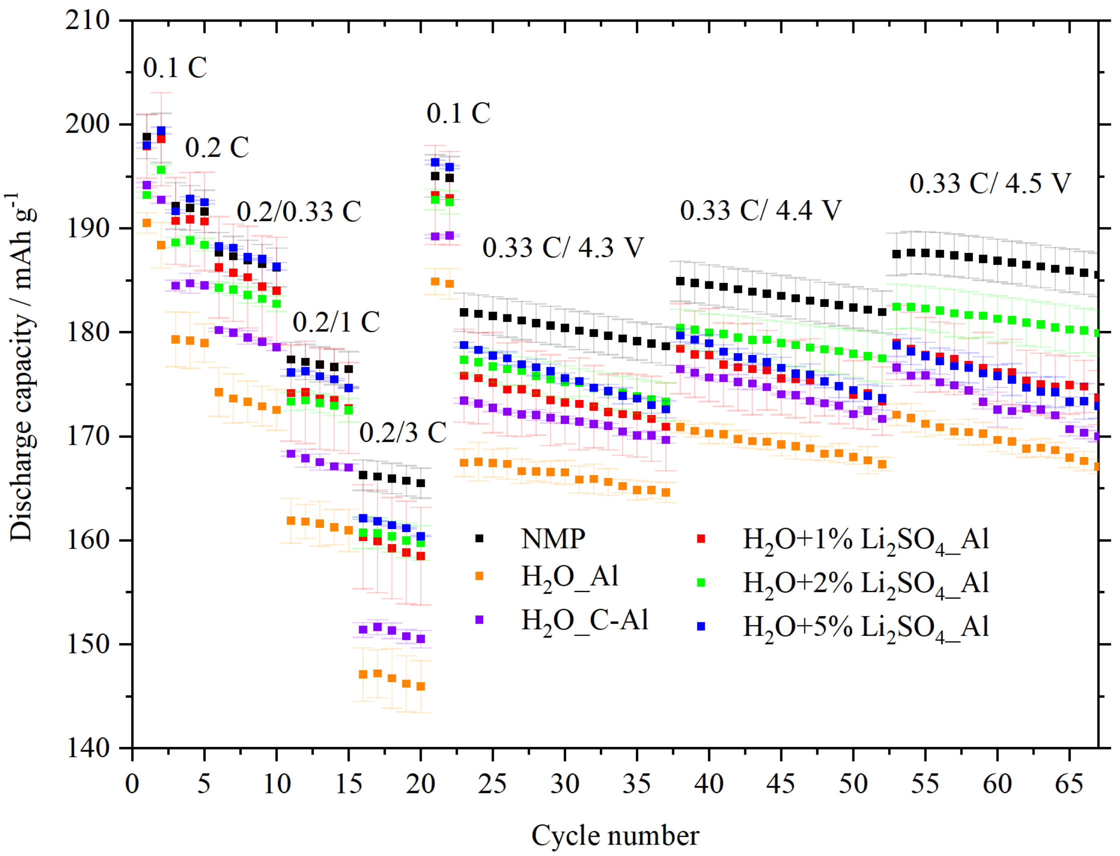

Figure S6: Electrochemical charge/discharge cycling performance of NCM811 || Li metal cells (two-electrode configuration) using NCM811 cathodes prepared by different processing methods in a cell voltage range of 2.9-4.3 V, 2.9-4.4 V or 2.9-4.5 V (1C=190 mA g^{-1}); Electrolyte: 1M $LiPF_6$ in EC:EMC 3:7 + 2 wt.%VC. Error bars represent the standard deviation of three cells per type of sample.

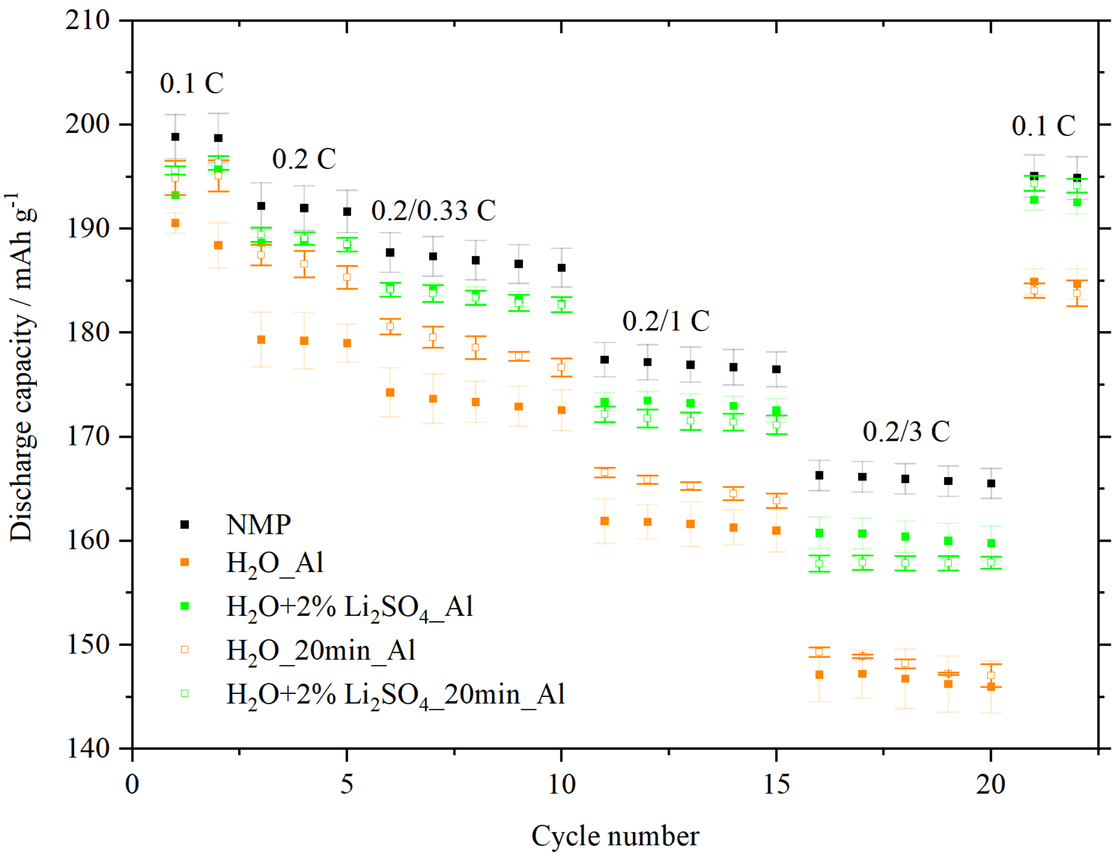

Figure S7: Electrochemical charge/discharge cycling performance of NCM811 || Li metal cells (two-electrode configuration) using NCM811 cathodes prepared with different dispersion time (60 vs. 20 min) in a cell voltage range of 2.9-4.3 V, 2.9-4.4 V or 2.9-4.5 V (1C=190 mA g^{-1}); Electrolyte: 1M $LiPF_6$ in EC:EMC 3:7 + 2 wt.%VC. Error bars represent the standard deviation of three cells per type of sample.

Table S 2: Electrochemical characteristics of NCM811 || graphite full-cells using NCM811 cathodes prepared by different processing methods. Cell voltage range: 2.8 - 4.2 V, N/P-ratio: 1.15:1.00, electrolyte: 1 M $LiPF_6$ in 3:7 vol. % EC/EMC + 2 wt.% VC.

Cathode	1st cycle discharge capacity at 0.1C / mAh g^{-1}	1st cycle Coulombic efficiency / %	5st cycle discharge capacity at 1C / mAh g^{-1}	SOH after 400 cycles / %
NMP	192	86.5	172	84
H_2O_Al	189	84.5	169	n.a.
H_2O_C-Al	189	85.5	165	83
H_2O+1% Li_2SO_4_Al	187	84.5	169	82
H_2O+2% Li_2SO_4_Al	189	85.6	170	84
H_2O+2% Li_2SO_4_C-Al	191	85.9	173	86
_H_2O+5% Li_2SO_4_ Al	192	84.2	169	81

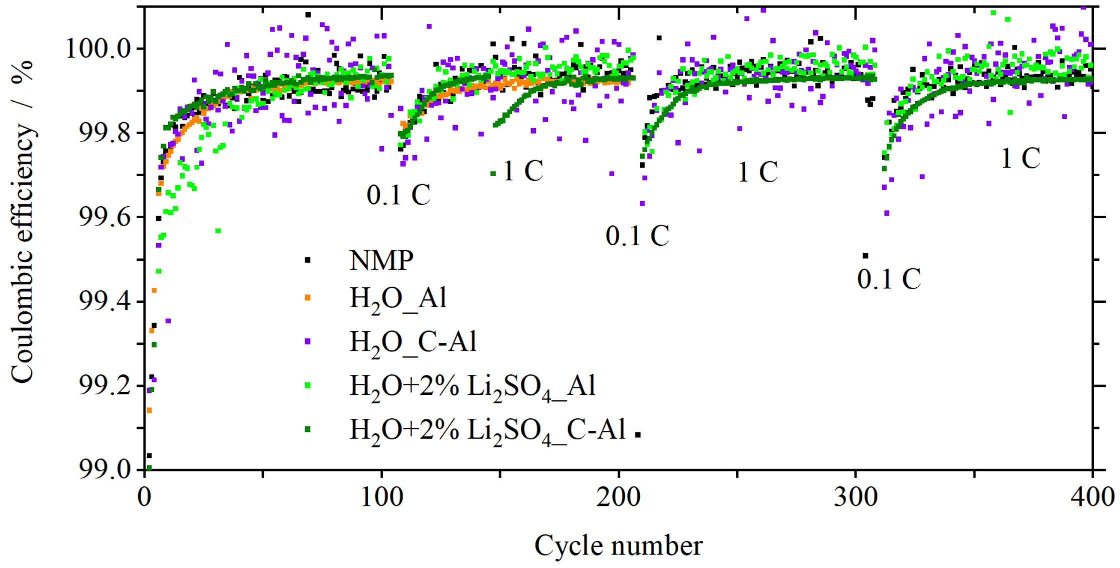

Figure S 8: Coulombic efficiencies of long-term cycling stability experiments of NCM811 || graphite full-cells using NCM811 cathodes prepared by different processing methods at a rate of 1C. Cell voltage range: 2.8 - 4.2 V, N/P-ratio: 1.15:1.00, electrolyte: 1 M $LiPF_6$ in 3:7 vol. % EC/EMC + 2 wt.% VC.

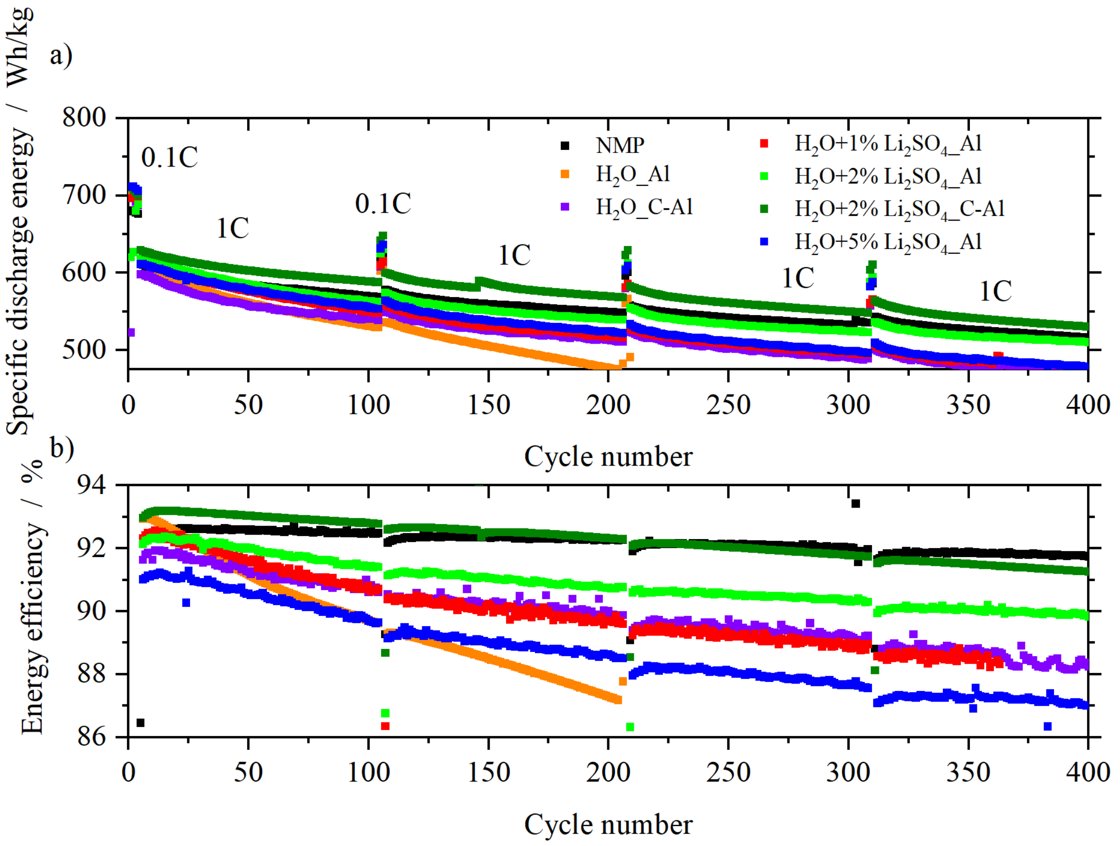

Figure S9: (a) Specific discharge energy and (b) energy efficiency of long-term cycling stability experiments of NCM811 || graphite full-cells using NCM811 cathodes prepared by different processing methods at a rate of 1C. Cell voltage range: 2.8 - 4.2 V, N/P-ratio: 1.15:1.00, electrolyte: 1 M $LiPF_6$ in 3:7 vol. % EC/EMC + 2 wt.% VC. The specific discharge energies on material level were calculated by multiplying the average discharge voltage (Figure 6a) with the discharge capacity (Figure 5a). Energy efficiencies are calculated by dividing the discharge energy and charge energy, respectively.

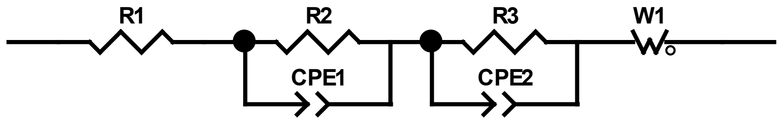

Figure S10: Equivalent circuit model for fitting the Electrochemical Impedance Spectroscopy (EIS) results of NCM811 || graphite full-cells. By using series-connected resistor (R1-R3) with parallel connected constant phase elements (CPE1 and CPE2) as circuit elements the impedances R_{EI} and R_{CT} can be described as Helmholtz double layers. The mass transfer is simulated by the Warburg diffusion (W).

References

[1] J. Sicklinger, M. Metzger, H. Beyer, D. Pritzl, H.A. Gasteiger, Ambient Storage Derived Surface Contamination of NCM811 and NCM111: Performance Implications and Mitigation Strategies, *Journal of The Electrochemical Society*, 166 (**2019**) A2322-A2335.
[2] M. Hofmann, M. Kapuschinski, U. Guntow, G.A. Giffin, Implications of Aqueous Processing for High Energy Density Cathode Materials: Part II. Water-Induced Surface Species on LiNi0.8Co0.15Al0.05O2, *Journal of The Electrochemical Society*, 167 (**2020**) 140535.
[3] A.C. Martinez, S. Grugeon, D. Cailleu, M. Courty, P. Tran-Van, B. Delobel, S. Laruelle, High reactivity of the nickel-rich LiNi1-x-yMnxCoyO2 layered materials surface towards H2O/CO2 atmosphere and LiPF6-based electrolyte, *Journal of Power Sources*, 468 (**2020**) 228204.
[4] E. Jakab, E. Mészáros, J. Borsa, Effect of slight chemical modification on the pyrolysis behavior of cellulose fibers, *Journal of Analytical and Applied Pyrolysis*, 87 (**2010**) 117-123.
[5] B. Song, F. Wu, Y. Zhu, Z. Hou, K.-s. Moon, C.-P. Wong, Effect of polymer binders on graphene-based free-standing electrodes for supercapacitors, *Electrochimica Acta*, 267 (**2018**) 213-221.
[6] N. Loeffler, J. von Zamory, N. Laszczynski, I. Doberdo, G.-T. Kim, S. Passerini, Performance of LiNi1/3Mn1/3Co1/3O2/graphite batteries based on aqueous binder, *Journal of Power Sources*, 248 (**2014**) 915-922.
